# Prevalence of Anxiety-Depressive Disorders and Stress Among Administrative Staff

**DOI:** 10.1192/j.eurpsy.2026.11430

**Published:** 2026-07-31

**Authors:** K. Imene, R. Nakhli, A. Asma, C. Sridi, C. Farah, F. Imen, B. Narjess, M. Maoua, M. Nejib

**Affiliations:** 1 Occupational Medicine Department, Farhat Hached University Hospital; 2 Faculty of Medicine of Sousse, University of Sousse; 3 Laboratoire de Recherche (LR19SP03); 4 Occupational Medicine Department, Sahloul University Hospital, Sousse, Tunisia

## Abstract

**Introduction:**

Tobacco use remains a major global public health challenge, causing more than 8 million deaths annually. Its association with anxiety and depressive disorders is well established, creating a vicious cycle in which smoking, often perceived as a stress reliever, paradoxically worsens psychological health. Administrative staff, although not exposed to significant physical occupational hazards, face specific constraints (mental workload, sedentary lifestyle, hierarchical pressures) that may promote such comorbidities.

**Objectives:**

To assess the prevalence of anxiety, depression, and perceived stress in smoking administrative employees.

**Methods:**

A cross-sectional descriptive observational study was conducted among 42 administrative employees who smoke at STEG. Data were collected using a self-administered questionnaire including sociodemographic characteristics, the Hospital Anxiety and Depression Scale (HADS), and the Perceived Stress Scale-10 (PSS-10).

**Results:**

The prevalence of smoking was 31.81% among administrative staff, with a mean tobacco exposure of 21 pack-years. The study population was predominantly male (95.2%), with a mean age of 44.36 ± 9.81 years, and 66.7% held higher education degrees. Anxiety and depressive disorders affected 76% of smokers (mean HADS score: 17), while 85.7% reported moderate to high stress levels (mean PSS score: 21).

**Conclusions:**

Our findings highlight the need for a multidisciplinary approach incorporating targeted prevention, early screening, and organizational interventions. Occupational health strategies should be redefined beyond traditional physical hazards to address psychosocial dimensions as a major public health concern.

**Disclosure of Interest:**

None Declared

